# The Prognostic Value of Serum Cytokines in Patients with Acute Ischemic Stroke

**DOI:** 10.14336/AD.2018.0820

**Published:** 2019-06-01

**Authors:** Xianmei Li, Siyang Lin, Xiaoli Chen, Wensi Huang, Qian Li, Hongxia Zhang, Xudong Chen, Shaohua Yang, Kunlin Jin, Bei Shao

**Affiliations:** ^1^Department of Rehabilitation, Wenzhou People’s Hospital, Wenzhou, China; ^2^Department of Neurology, The First Affiliated Hospital of Wenzhou Medical University, Wenzhou, China; ^3^Department of Neurology, The People’s Hospital of Pingyang, Wenzhou, China; ^4^Department of Neurology, Jinhua Municipal Central Hospital, Wenzhou, China; ^5^Department of Pharmacology and Neuroscience, University of North Texas Health Science Center, Fort Worth, Texas, USA

**Keywords:** acute ischemic stroke, cytokines, inflammation, prognosis, stroke severity

## Abstract

The inflammatory response is an unavoidable process and contributes to the destruction of cerebral tissue during the acute ischemic stroke (AIS) phase and has not been addressed fully to date. Insightful understanding of correlation of inflammatory mediators and stroke outcome may provide new biomarkers or therapeutic approaches for ischemic stroke. Here, we prospectively recruited 180 first-ever AIS patients within 72 hrs after stroke onset. We used the National Institutes of Health Stroke Scale (NIHSS) to quantify stroke severity and modified Rankin scale (mRS) to assess the 3-month outcome for AIS patients. Initially, we screened 35 cytokines, chemokines, and growth factors in sera from 75 AIS patients and control subjects. Cytokines that were of interest were further investigated in the 180 AIS patients and 14 heathy controls. We found that IL-1RA, IL-1β, IL-4, IL-5, IL-6, IL-7, IL-9, IL-10, IL-13, IL-15, EGF, G-CSF, Flt-3L, GM-CSF and Fractalkine levels were significantly decreased in severe stroke patients. In particular, IL-1β, IL-4, IL-5, IL-7, IL-9, IL-10, IL-15, G-CSF and GM-CSF were significantly reduced in AIS patients with poor outcome, compared to those with good prognosis. IL-6 was notably higher in the poor outcome group. Only IL-9 level decreased in the large infarct volume group. After adjusting for confounders, we found that IL-5 was an independent protective factor for prognosis in AIS patients with an adjusted OR of 0.042 (P = 0.007), whereas IL-6 was an independent risk predictor for AIS patients with an adjusted OR of 1.293 (P = 0.003). Our study suggests the levels of serum cytokines are related to stroke severity, short-term prognosis and cerebral infarct volume in AIS patients.

Cerebrovascular disease, a global health problem, has become one of the major cause of adult mortality and disability worldwide, and ranks second only to ischemic heart disease [1-3]. Acute ischemic stroke (AIS), caused by cerebral embolism or arterial thrombosis, accounts for 80-85% of cerebrovascular disease and is the major subtype of all strokes [4]. Inflammation following AIS is considered an inevitable pathological process involved in post-ischemic injury in the brain [5, 6]. After the initial injury, a series of detrimental secondary events occur and the blood-brain barrier (BBB) becomes damaged. Activated peripheral immune cells including neutrophils and T-cells can then cross the BBB and accumulate at the site of injury [5]. These cascade reactions would indeed aggravate cerebral infarction, which potentially contributes to the destruction of cerebral tissue during the AIS phase [7, 8]. On the other hand, an increasing number of reports have showed that the inflammatory response after stroke plays a critical role for functional recovery in the later stages [9, 10]. A probable reason for this phenomenon can be attributed to dynamic alterations in the release of several pro- and anti-inflammatory cytokines in the brain that could affect the progression of cerebral infarction [11]. These chemokines are mainly produced by resident microglial cells and infiltrating immune cells, which could attract and activate leukocytes [12, 13]. Some chemokines are even capable of recruiting nonimmune cells like neural stem cells (NSCs), neural progenitor cells (NPCs), endothelial cells and bone marrow stromal cells (BMSCs) to the lesion site which may exert beneficial impact on protection and recovery [14].

Th2 type cytokines including interleukin-4 (IL-4) and interleukin-5 (IL-5) were found to play beneficial roles in the repair of brain damage, suppress post-stroke inflammation, and have the capability to induce neurotrophic factors in astrocytes [15-17]. Interleukin-6 (IL-6), well-known for its pro-inflammatory function also possess neurotrophic and regenerative capabilities after cerebral ischemia [18, 19]. Interleukin-7 (IL-7) is a pleiotropic cytokine with multiple effects. Arya *et al*. reported that IL-7 could enhance the expression of monocyte chemoattractant protein-1 (MCP-1) in patients with unstable angina, and was associated with hyperlipidemia and atherosclerosis [20]. Interleukin-9 (IL-9) is a pro-inflammatory cytokine, secreted by Th9 cells. Interleukin-10 (IL-10) is generally viewed as an anti-inflammatory cytokine that helps to restrain pro-inflammatory cytokines and depress cytokine receptor expression and receptor activation [21]. Interleukin-13 (IL-13) is a mediator of allergic inflammation and different diseases including asthma [22]. Interleukin-15 (IL-15) mainly regulates the activation and proliferation of T and natural killer (NK) cells. Fractalkine (CX3CL1), macrophage-derived chemokine (MDC, CCL22) and macrophage inflammatory protein-1 alpha (MIP-1α, CCL3) are members of chemokine family, which serve different functions in inflammation, such as guiding the migration of immune cells or regulating activation and maturation of cells. Fms-like tyrosine kinase-3 ligand (Flt-3L), epidermal growth factor (EGF), granulocyte-colony stimulating factor (G-CSF), granulocyte-macrophage colony-stimulating factor (GM-CSF) are growth factors. Therefore, these inflammatory mediators, cytokines in particular, have been considered as biomarkers for stroke pathogenesis and prognosis. Previous reports characterized an acute immune response to ischemic stroke by profiling certain cytokines and chemokines (e.g., IL-1α and β, IL-6, IL-8, IL-9, IL-10, IL-12, IL-18, TNFα and soluble TNF-receptors p55, p75 and GRO-α) in the sera or cerebrospinal fluid of stroke patients [23-25]. However, various known cytokines have been studied in limited number thereby restricting the significance of predictive value of these biomarkers in patient outcome after AIS.

To determine the significance of serum inflammatory cytokines in patient outcome after acute ischemic stroke, we simultaneously measured 35 cytokines, chemokines, and growth factors in a single patient group using a multiplex immunoassay and found that the levels of inflammatory mediators in the serum can be used to deduce stroke severity, short-term prognosis and cerebral infarct volume.

## MATERIALS AND METHODS

### Study population

One hundred and eighty patients with first-ever AIS were enrolled at the Department of Neurology, the First Affiliated Hospital of Wenzhou Medical University, from April 2014 to September 2016. All AIS patients within 72 hrs after stroke onset were selected based on the criteria set out by the World Health Organization [26]. Etiology subtypes of ischemic stroke were classified according to the criteria of Trial of Org 10172 in Acute Stroke Treatment (TOAST) [27]. The exclusion criteria for first-ever AIS patients are: (i) history of any serious central nervous system disease, such as Parkinson’s disease, craniocerebral trauma, dementia, hematencephalon, cerebral infarction or subarachnoid hemorrhage; (ii) failure of important organs, such as heart failure, severe liver or renal insufficiency; (iii) autoimmune disease (AID) or the use of steroids or immunosuppressants; (iv) a history of cancer; (v) a serious history of infection, trauma or surgery within 4 weeks prior to onset.

The study followed ethical guidelines and obtained the approval of the ethics committee at the First Affiliated Hospital of Wenzhou Medical University. Patient consent forms were signed by each patient or their relatives before inclusion.

### Data collection

The following basic clinical information of all subjects during the first 24 hrs after onset of stroke were collected by well-trained neurologists: gender, age, smoking habit, alcohol abuse, hypertension, diabetes mellitus (DM), hyperlipemia, cardiovascular diseases, systolic blood pressure (SBP) and diastolic blood pressure (DBP) on admission and biochemical indices. The National Institutes of Health Stroke Scale (NIHSS) score was used to measure the severity of AIS patients by the neurologist within 24 hrs of admission and discharge [28, 29]. The minor stroke was defined as NIHSS score < 5 according to previous studies [30, 31]. The short-term functional outcome was assessed by telephone interviews or outpatient service at 3 months after stroke onset using the modified Rankin scale (mRS, scores range from 0 to 6) [32]. A poor functional outcome was defined as mRS score 3-6, while favorable outcome was defined as mRS score 0-2 [33, 34].

**Table 1:** Baseline characteristics of AIS patients with favorable or poor outcomes.

Characteristics	Total (N= 167)	Prognosis at 3 months follow-up		P value
		Favorable Outcome (N= 113)	Poor Outcome (N=54)	
Age (years)	63.02 ± 9.84	62.08 ± 10.35	65.00 ± 8.43	0.073
Males (%)	100 (60.0)	73 (64.6)	27 (50.0)	0.073
SBP (mmHg)	162.34 ± 24.41	160.18 ± 22.76	166.87 ± 27.20	0.097
DBP (mmHg)	85.43 ± 13.68	85.48 ± 13.13	85.31 ± 14.90	0.943
Hypertension (%)	141 (84.4)	93 (82.3)	48 (88.9)	0.273
Hyperlipidemia (%)	32 (19.2)	21 (18.6)	11 (20.4)	0.784
Diabetes (%)	55 (32.9)	33 (29.2)	22 (40.7)	0.139
Cardiac disease (%)	19 (11.4)	10 (8.8)	9 (16.7)	0.138
Smoking (%)	57 (34.1)	31 (27.4)	24 (44.4)	0.053
Alcohol drinking (%)	43 (25.7)	33 (29.2)	9 (16.7)	0.064
Stroke etiologic subtypes (%)				0.982
Large-artery atherosclerosis	110 (65.7)	76 (67.3)	34 (62.3)	-
Cardioembolic	13 (7.8)	8 (7.1)	5 (9.3)	-
Small-vessel disease	38 (22.8)	28 (24.8)	10 (18.5)	-
Other or unknown cause	6 (3.6)	2 (1.8)	4 (7.4)	-
BMI (kg/m^2^)	23.76 ± 3.14	23.72 ± 3.11	23.84 ± 3.23	0.808
Laboratory tests				
WBC (10^9^/L)	6.35 (5.57 - 7.79)	6.29 (5.44 - 7.58)	6.51 (5.82 - 8.60)	0.081
Neutrophils (10^9^/L)	3.88 (3.03 - 4.92)	3.70 (2.89 - 4.82)	4.22 (3.38 - 5.74)	0.009
Hs-CRP (mmol/L)	1.86 (0.87 - 3.91)	1.67 (0.60 - 3.30)	2.64 (1.37 - 4.90)	0.027
IL-1RA (pg/mL)	2.21 (0.68 - 8.66)	2.30 (0.97 - 7.710)	1.4 (0.27 - 5.56)	0.165
IL-1α (pg/mL)	2.96 (0.32 - 17.69)	2.96 (0.32 - 17.69)	1.21 (0.08 - 27.21)	0.365
IL-1β (pg/mL)	1.06 (0.73 - 1.60)	1.11 (0.82 - 1.60)	0.87 (0.60 - 1.56)	0.007
IL-4 (pg/mL)	1.82 (0.54 - 6.47)	3.10 (1.13 - 8.15)	0.53 (0.28 - 1.32)	< 0.001
IL-5 (pg/mL)	0.58 (0.35 - 0.82)	0.63 (0.48 - 0.86)	0.32 (0.24 - 0.66)	< 0.001
IL-6 (pg/mL)	1.17 (0.65 - 1.95)	1.17 (0.68 - 1.90)	2.06 (0.49 - 2.13)	0.021
IL-7 (pg/mL)	1.62 (0.86 - 2.5)	1.78 (1.31 - 2.67)	0.77 (0.45 - 1.95)	< 0.001
IL-8 (pg/mL)	4.59 (3.03 - 9.17)	4.59 (3.11 - 7.16)	5.34 (2.86 - 14.43)	0.262
IL-9 (pg/mL)	0.8 (0.36 - 1.15)	0.95 (0.70 - 1.19)	0.31 (0.24 - 0.73)	< 0.001
IL-10 (pg/mL)	0.87 (0.60 - 1.80)	0.96 (0.65 - 2.13)	0.74 (0.49 - 1.22)	0.027
IL-13 (pg/mL)	0.05 (0.02 - 0.17)	0.06 (0.02 - 0.16)	0.03 (0.01 - 0.15)	0.071
IL-15 (pg/mL)	1.3 (0.77 - 1.77)	1.37 (0.94 - 1.98)	0.94 (0.54 - 1.65)	0.002
EGF (pg/mL)	3.92 (0.84 - 20.06)	5.96 (1.04 - 20.59)	1.81 (0.26 - 17.69)	0.084
G-CSF (pg/mL)	8.64 (4.27 - 17.69)	10.77 (5.68 - 16.06)	5.20 (0.20 - 18.61)	0.017
Flt-3 (pg/mL)	0.4 (0.07 - 2.26)	0.47 (0.08 - 2.53)	0.25 (0.03 - 1.60)	0.44
GM-CSF (pg/mL)	4.27 (1.19 - 20.61)	7.71 (2.13 - 26.46)	1.51 (0.83 - 6.47)	< 0.001
Fractalkine (pg/mL)	19.57 (3.62 - 59.16)	17.99 (7.06 - 56.99)	21.75 (0.07 - 60.03)	0.225
IFN-γ (pg/mL)	4.7 (2.06 - 9.17)	4.54 (2.19 - 7.75)	3.76 (1.43 - 13.67)	0.897
MDC (pg/mL)	335.44 (248.06 - 471.03)	337.56 (246.52 - 469.62)	352.74 (251.20 - 540.90)	0.365
MIP-1α (pg/mL)	2.03 (1.43 - 3.8)	2.03 (1.45 - 3.08)	2.39 (1.13 - 7.64)	0.485
Infarct volume (cm^3^)	1.26 (0.40 - 3.42)	0.86 (0.29 - 2.06)	3.07 (1.02 - 5.93)	< 0.001
NIHSS score on admission, median (IQR)	3.00 (2.00 - 5.00)	3.00 (1.00 - 4.00)	7.00 (4.00 - 10.00)	< 0.001
NIHSS score on discharge, median (IQR)	3.00 (1.00 - 5.00)	2.00 (1.00 - 3.00)	6.00 (4.75 - 9.00)	< 0.001
Medication, (%)				
Statin	135 (80.8)	99 (87.6)	36 (66.7)	0.002
Anticoagulation agents	8 (4.8)	6 (5.3)	2 (3.7)	0.448
Antiplatelet agents	132 (79.0)	97 (85.8)	35 (64.8)	0.001

Abbreviations: SBP, systolic blood pressure; DBP, diastolic blood pressure; BMI, body mass index; WBC, leukocyte; Hs-CRP, High-sensitivity C-reactive protein; IL-1RA, interleukin-1 receptor antagonist; IL-1α, interleukin 1 alpha; IL-1β, interleukin 1 beta; IL-4, interleukin-4; IL-5, interleukin-5; IL-6, interleukin-6; IL-7, interleukin-7; IL-8, interleukin-8; IL-9, interleukin-9; IL-10, interleukin-10; IL-13, interleukin-13; IL-15, interleukin-15; Flt-3, Fms-like tyrosine kinase 3; EGF, epidermal growth factor ; G-CSF, granulocyte-colony stimulating factor; GM-CSF, granulocyte-macrophage colony-stimulating factor; MDC, macrophage-derived chemokine; MIP-1α, macrophage inflammatory protein-1 alpha; IQR, interquartile range; NIHSS, National Institutes of Health Stroke Scale.

All participants were examined using Cranial Magnetic Resonance Imaging (MRI) scans. The infarction size in diffusion-weighted imaging (DWI) was measured using G3PACS software by two neuroradiologists independently. Firstly, we selected the slice with the largest lesion by eye and the longest lesion axis (A axis) on this slice was measured. A second line (B axis) was drawn perpendicular to the first at the widest dimension. A third axis, the z (C) axis, was computed by multiplying the number of slices by slice thickness (7 mm). The formula was 0.5 x A x B x C [35]. According to a previous study, a small infarct volume was defined as less than 5 cm^3^, while a large infarct volume is larger than 5 cm^3^ [36].

**Figure 1.**
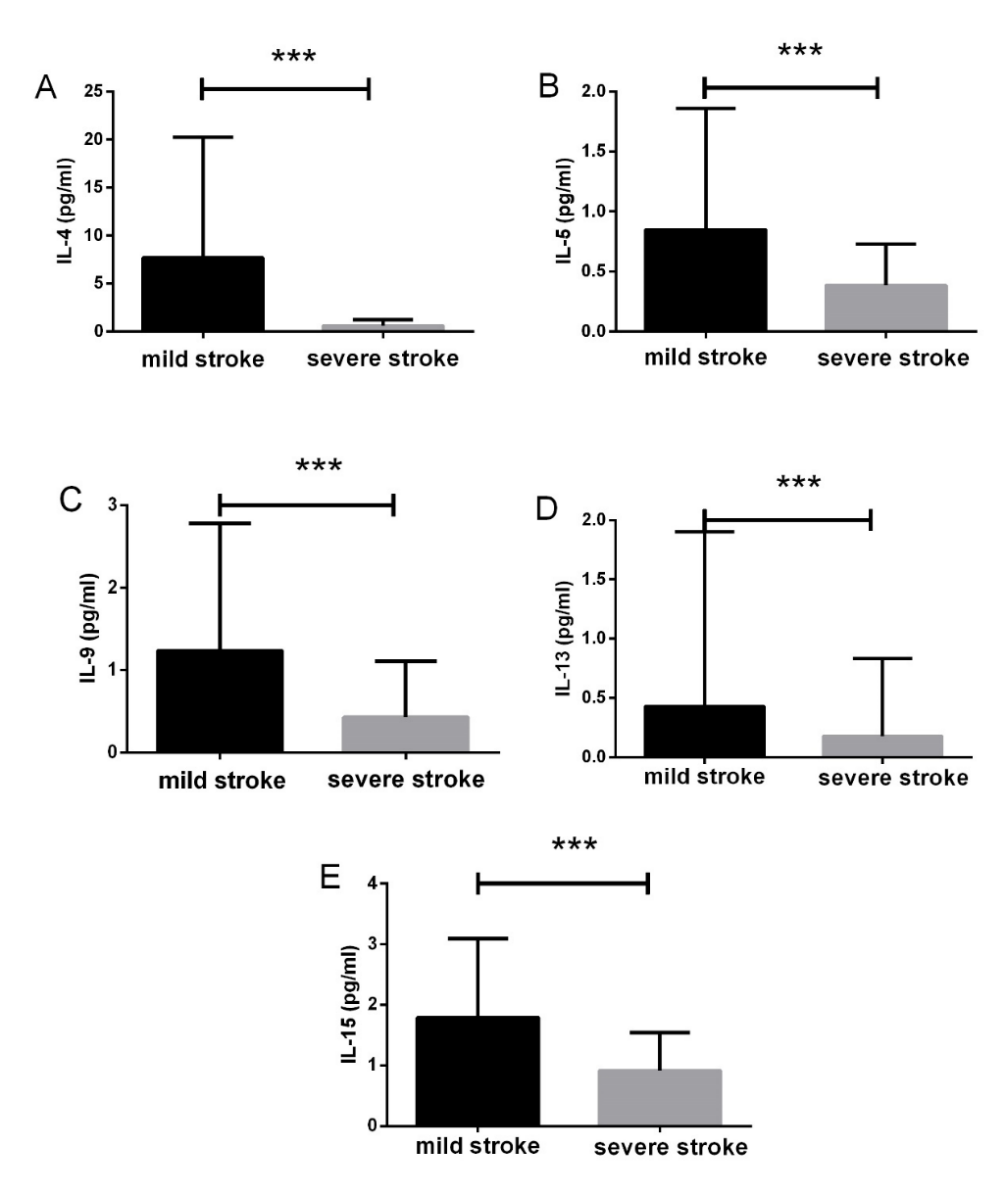
The relationship between various inflammatory cytokines and stroke severity The levels of IL-4, IL-5, IL-9, IL-13 and IL-15 were significantly decreased in the severe stroke group compared with the minor stroke group. Data are presented as mean ± SD, ***P < 0.001.

**Table 2:** Levels of serum cytokines in different groups of stroke severity.

	Median (IQR)			
Cytokines	Minor Stroke Group (N=53)	Severe Stroke Group (N=94)	P value	Control (N=14)
Hs-CRP (mmol/L)	1.55 (0.59 - 3.50)	2.56 (1.39 - 4.81)	0.019	—
IL-1RA (pg/mL)	2.56 (1.35 - 9.45)	1.94 (0.48 - 8.15)	0.028	0.40(0.07-6.38)
IL-1α (pg/mL)	3.2 (0.35 - 14.43)	1.93 (0.19 - 24.28)	0.234	0.045 (0.02 - 28.49)
IL-1β (pg/mL)	1.11 (0.92 - 1.68)	0.93 (0.61 - 1.51)	< 0.001	0.73 (0.53 - 2.23)
IL-4 (pg/mL)	3.62 (1.48 - 9.31)	0.96 (0.32 - 3.10)	< 0.001	1.03 (0.44 - 5.45)
IL-5 (pg/mL)	0.70 (0.51 - 0.90)	0.40 (0.24 - 0.70)	< 0.001	0.23 (0.18 - 0.42)
IL-6 (pg/mL)	1.34 (0.80 - 2.32)	1.00 (0.49 - 1.76)	< 0.001	0.70 (0.33 - 1.36)
IL-7 (pg/mL)	2.10 (1.45 - 2.84)	1.09 (0.47 - 2.06)	< 0.001	0.82 (0.44 - 2.72)
IL-8 (pg/mL)	4.76 (3.39 - 8.45)	4.09 (2.38 - 11.73)	0.238	21.75 (2.05 - 82.14)
IL-9 (pg/mL)	1.01 (0.77 - 1.29)	0.37 (0.25 - 0.90)	< 0.001	0.33 (0.26 - 0.76)
IL-10 (pg/mL)	1.12 (0.69 - 2.14)	0.70 (0.49 - 1.27)	< 0.001	0.41 (0.23 - 1.16)
IL-13 (pg/mL)	0.075 (0.03 - 0.22)	0.03 (0.01 - 0.11)	< 0.001	0.02 (0.01 - 0.31)
IL-15 (pg/mL)	1.42 (1.07 - 2.13)	0.93 (0.59 - 1.65)	< 0.001	0.83 (0.55 - 1.38)
EGF (pg/mL)	6.69 (1.35 - 22.78)	2.14 (0.36 - 17.05)	0.016	17.83 (1.17 - 53.23)
G-CSF (pg/mL)	12.24 (7.00 - 19.82)	5.73 (0.22 - 14.05)	< 0.001	1.15 (0.27 - 9.91)
Flt-3 (pg/mL)	0.52 (0.10 - 2.83)	0.25 (0.04 - 1.60)	0.007	0.56 (0.10 - 23.93)
GM-CSF (pg/mL)	11.56 (2.42 - 41.66)	1.85 (0.91 - 9.17)	< 0.001	1.17 (0.86 - 5.27)
Fractalkine (pg/mL)	23.93 (7.69 - 73.27)	15.60 (0.20 - 50.88)	< 0.001	6.47 (0.20 - 23.93)
IFN-γ (pg/mL)	5.14 (2.53 - 7.98)	3.58 (1.53 - 11.00)	0.068	2.71 (1.39 - 11.44)
MDC (pg/mL)	333.35 (246.52 - 462.26)	352.74 (249.64 - 540.90)	0.123	731.67 (511.10 - 923.05)
MIP-1α (pg/mL)	2.18 (1.54 - 3.36)	1.75 (1.26 - 4.95)	0.754	5.23 (0.85 - 28.96)

Abbreviations: SBP, systolic blood pressure; DBP, diastolic blood pressure; BMI, body mass index; WBC, leukocyte; Hs-CRP, High-sensitivity C-reactive protein; IL-1RA, interleukin-1 receptor antagonist; IL-1α, interleukin 1 alpha; IL-1β, interleukin 1 beta; IL-4, interleukin-4; IL-5, interleukin-5; IL-6, interleukin-6; IL-7, interleukin-7; IL-8, interleukin-8; IL-9, interleukin-9; IL-10, interleukin-10; IL-13, interleukin-13; IL-15, interleukin-15; Flt-3, Fms-like tyrosine kinase 3; EGF, epidermal growth factor ; G-CSF, granulocyte-colony stimulating factor; GM-CSF, granulocyte-macrophage colony-stimulating factor; MDC, macrophage-derived chemokine; MIP-1α, macrophage inflammatory protein-1 alpha; IQR, interquartile range; NIHSS, National Institutes of Health Stroke Scale.

### Cytokine measurement

Blood samples of all participants were collected within 24 hrs after admission. The serum levels of cytokines, chemokines, and growth factors were measured using human cytokine/chemokine magnetic bead panel kit (HCYTMAG-60K-PX38, EMD Millipore, Germany). All procedures were performed according to the manufacturer’s instructions and data were analyzed with the xPONENT software. Other laboratory parameters included white blood cell (WBC) count, neutrophil and high-sensitivity C-reactive protein (Hs-CRP) were tested in the hospital’s central biochemistry laboratory.

### Statistical analysis

All statistical analyses were performed with SPSS version 23.0 (Chicago, IL) and statistical significance was set at P < 0.05. The Kolmogorov-Smirnov (K-S) test was applied to assess the normality of continuous variables. Continuous variables of a normal distribution were expressed as the mean value ± standard deviation (SD) and analyzed using the unpaired *t*-test. Non-normally distributed variables were expressed as medians with inter-quartile ranges (IQR) and analyzed using the Mann-Whitney U test. Meanwhile, the Chi-Squared test was used to compare frequency and percentage in categorical variables. Spearman’s rank correlation or Pearson correlation was performed for bivariate correlation between NIHSS scores, infarct volume and serum markers. The relationship between serum markers and 3-months functional outcome of AIS patients was analyzed by multivariate logistic regression analysis after adjusting for confounders. The receiver operating characteristic (ROC) curve was used to determine the predictive values of the serum levels of these inflammatory markers to predict short-term prognosis in AIS patients. In addition, area under the curve (AUC) was computed as a measurement of the accuracy of the data.

**Figure 2.**
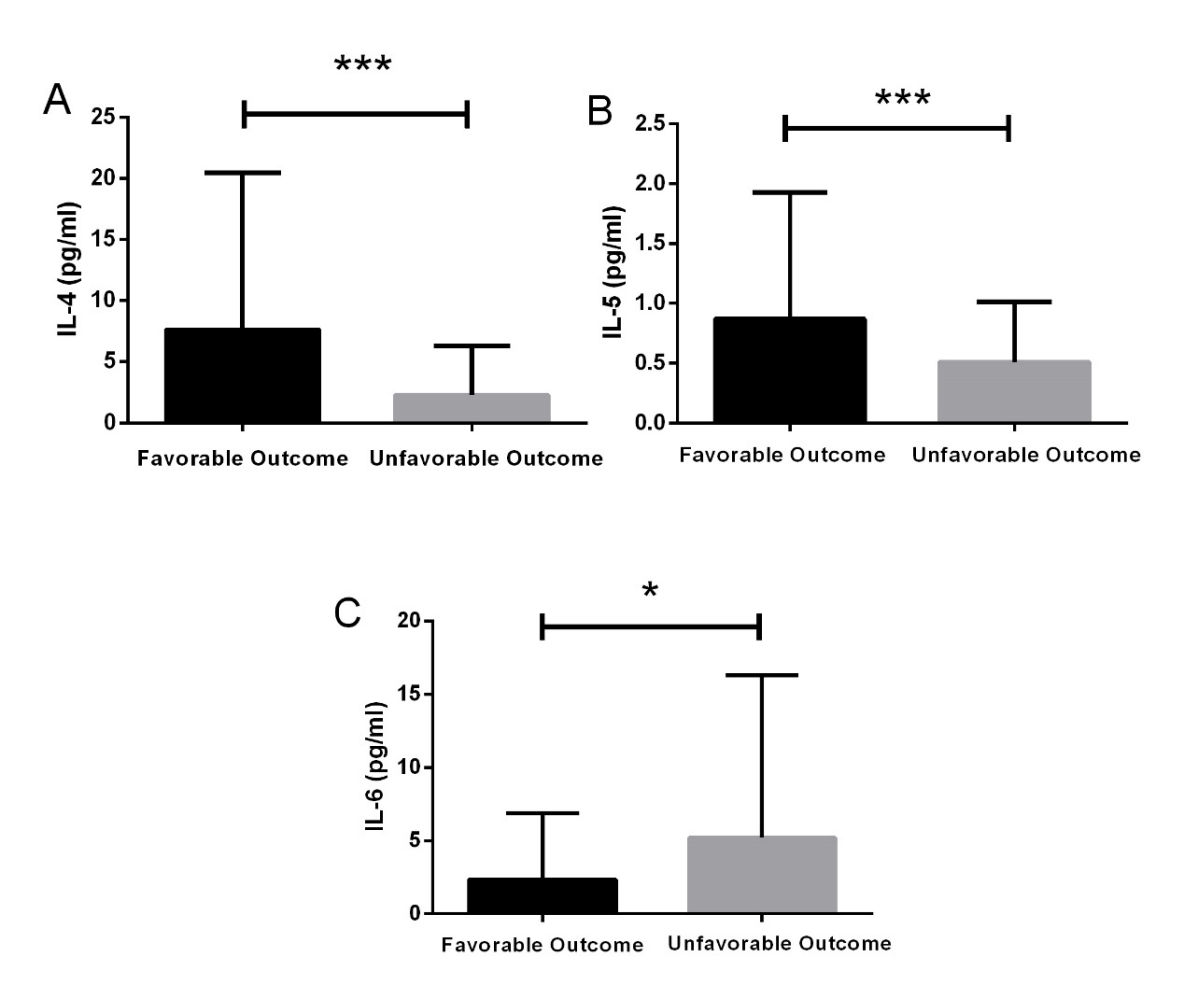
The relationship between various inflammatory cytokines and stroke outcomes The levels of IL-4 and IL-5 were significantly increased in the favorable outcome group compared with the poor outcome group. However, the concentration of IL-6 was significantly decreased in the favorable outcome group. Data are presented as mean ± SD, *P < 0.05, ***P < 0.001.

## RESULTS

### Baseline patient characteristics

A total of 180 patients that met the inclusion criteria were recruited and 167 of them completed follow-up (13 patients could not be reached). The average age of AIS patients was 63.02 ± 9.84 years and 60% of AIS patients enrolled were male. The median (quartiles) NIHSS score on admission was 3 (IQR, 2 - 5) and on discharge was 3 (IQR, 1 - 5). Poor outcome at 3 months was found in 54 patients (32.3%) with the average age being 65.00 ± 8.43 years. The characteristics of AIS patients with good or poor 3-months outcomes are shown in Table 1.

### Levels of serum cytokines reveal the severity of stroke

We initially screened 35 inflammatory mediators in 75 patients, and found that IL-4, IL-7, IL-9, GM-CSF and MIP-1α were significantly decreased in the severe stroke group, compared with control group (P < 0.05; data not shown). AIS patients were then divided into two groups according to the NIHSS score on admission: 53 (31.7%) patients in the minor stroke group (NIHSS < 5) and 94 (68.3%) patients in the severe stroke group (NIHSS ≥ 5). The serum levels of IL-1RA, IL-1β, IL-4, IL-5, IL-6, IL-7, IL-9, IL-10, IL-13, IL-15, EGF, G-CSF, Flt-3L, GM-CSF, and Fractalkine levels were significantly lower in the severe versus (*vs.*) the minor stroke group (Fig. 1). Circulating levels of IL-1α, MDC, MIP-1α, and IFN-γ not altered (Table 2). Of note, the level of Hs-CRP was significantly increased in the severe-stroke group *vs.* the minor-stroke group.

**Table 3:** Logistic regression model with predictors of poor outcome (N=167).

Characteristics	Unadjusted OR (95% CI)	P value	Adjusted OR (95% CI)	P value
Age (years)	1.032 (0.997 - 1.069)	0.074	—	—
Male (%)	0.548 (0.284 - 1.058)	0.073	—	—
SBP (mmHg)	1.012 (0.998 - 1.026)	0.099	—	—
DBP (mmHg)	0.999 (0.976 - 1.023)	0.942	—	—
Hypertension (%)	1.720 (0.648 - 4.568)	0.276	—	—
Hyperlipidemia (%)	1.121 (0.496 - 2.530)	0.784	—	—
Diabetes (%)	1.667 (0.846 - 3.282)	0.139	—	—
Cardiac disease (%)	2.060 (0.784 - 5.414)	0.143	—	—
Smoking (%)	1.939 (0.990 - 3.800)	0.054	—	—
Alcohol drinking (%)	0.465 (0.204 - 1.056)	0.067	—	—
Stroke etiologic subtypes (%)	1.010 (0.699 - 1.460)	0.958	—	—
Large-artery atherosclerosis	—	—	—	—
Cardioembolic	—	—	—	—
Small-vessel disease	—	—	—	—
Other or unknown cause	—	—	—	—
BMI (kg/m2)	1.013 (0.913 - 1.123)	0.807	—	—
Laboratory tests				
WBC (10^9^ /L)	1.192 (0.998 - 1.423)	0.052	—	—
Neutrophils (10^9^ /L)	1.333 (1.075 - 1.654)	0.009	—	—
Hs-CRP (mmol/L)	1.008 (0.985 - 1.033)	0.484	—	—
IL-1RA (pg/ml)	1.004 (0.996 - 1.011)	0.342	—	—
IL-1α (pg/ml)	1.001 (0.998 - 1.004)	0.593	—	—
IL-1β (pg/ml)	0.992 (0.813 - 1.210)	0.937	—	—
IL-4 (pg/ml)	0.869 (0.788 - 0.960)	0.005	—	—
IL-5 (pg/ml)	0.244 (0.087 - 0.682)	0.007	0.039 (0.003 - 0.475)	0.011
IL-6 (pg/ml)	1.051(1.002 - 1.103)	0.041	1.329 (1.095 - 1.612)	0.004
IL-7 (pg/ml)	0.981 (0.909 - 1.059)	0.630	—	—
IL-9 (pg/ml)	0.768 (0.508 - 1.161)	0.211	—	—
IL-10 (pg/ml)	0.962 (0.845 - 1.094)	0.554	—	—
IL-13 (pg/ml)	1.052 (0.853 - 1.299)	0.634	—	—
IL-15 (pg/ml)	0.726 (0.514 - 1.025)	0.069	—	—
EGF (pg/ml)	1.003 (0.996 - 1.009)	0.421	—	—
G-CSF (pg/ml)	1.004 (0.998 - 1.009)	0.165	—	—
GM-CSF (pg/ml)	1.000 (0.994 - 1.007)	0.916	—	—
Flt-3 (pg/ml)	1.015 (0.992 - 1.038)	0.200	—	—
Fractalkine (pg/ml)	1.002 (0.999 - 1.005)	0.244	—	—
IFN-γ (pg/ml)	1.004 (0.996 - 1.013)	0.324	—	—
MDC (pg/ml)	1.001 (1.000 - 1.003)	0.071	—	—
MIP-1α (pg/ml)	1.069 (1.010 - 1.131)	0.021	—	—
Infract volume (cm^3^)	1.043 (0.997 - 1.090)	0.065	—	—
NIHSS score on admission, median (IQR)	2.148 (1.679 - 2.748)	<0.001	—	—
NIHSS score on discharge, median (IQR)	2.477 (1.855 - 3.307)	<0.001	2.494 (1.364 - 4.562)	0.003
Medications, no. (%)				
Statin	0.283 (0.128 - 0.627)	0.002	—	—
Anticoagulation agents	2.135 (0.293 - 5.576)	0.455	—	—
Antiplatelet agents	0.304 (0.141 - 0.656)	0.002	—	—

Abbreviations: SBP, systolic blood pressure; DBP, diastolic blood pressure; BMI, body mass index; WBC, leukocyte; Hs-CRP, High-sensitivity C-reactive protein; IL-1RA, interleukin-1 receptor antagonist; IL-1α, interleukin 1 alpha; IL-1β, interleukin 1 beta; IL-4, interleukin-4; IL-5, interleukin-5; IL-6, interleukin-6; IL-7, interleukin-7; IL-8, interleukin-8; IL-9, interleukin-9; IL-10, interleukin-10; IL-13, interleukin-13; IL-15, interleukin-15; Flt-3, Fms-like tyrosine kinase 3; EGF, epidermal growth factor ; G-CSF, granulocyte-colony stimulating factor; GM-CSF, granulocyte-macrophage colony-stimulating factor; MDC, macrophage-derived chemokine; MIP-1α, macrophage inflammatory protein-1 alpha; IQR, interquartile range; NIHSS, National Institutes of Health Stroke Scale.

### Levels of serum cytokines in relation to cerebral infarction volume

All AIS patients underwent cranial MRI scans and the median (quartiles) infarct volume was 1.26 (IQR, 0.40 - 3.42). 29 patients belonged to large infarct volume group (≥ 5cm^3^; median 3.07; IQR, 1.02 - 5.93) and 138 patients were in the small infarct volume group (< 5 cm^3^; median 0.86; IQR, 0.29 - 2.06). Serum Hs-CRP level was significantly higher in patients with large infarct volumes (P = 0.014), while IL-9 level increased significantly in the small infarct volume group (P = 0.034). Interestingly, serum IL-4 was found to be decreased in the large infarct volume group although it did not reach significance (P = 0.090) (Table 1).

**Figure 3.**
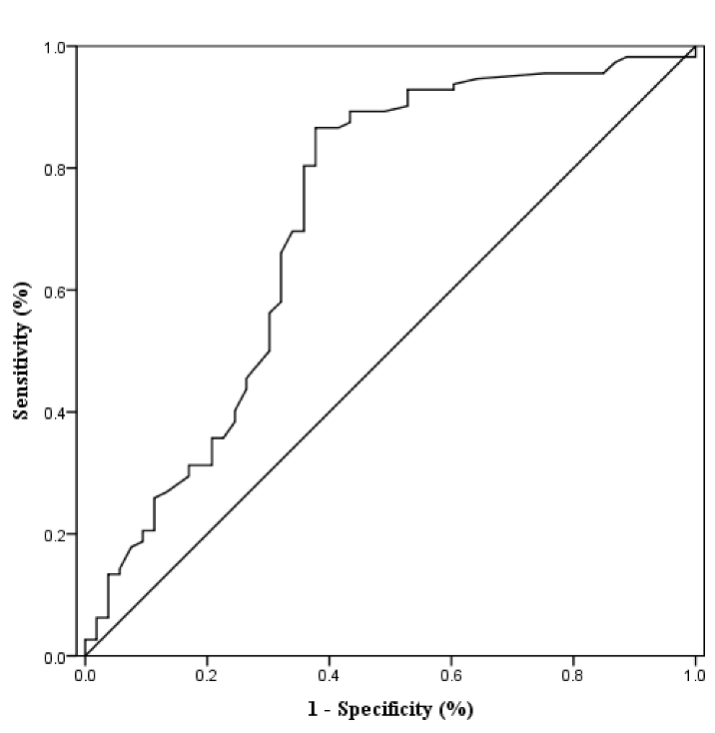
ROC curve of IL-5 for predicting 3-months outcome of AIS patients The optimal cutoff value was 0.385 pg/mL with a sensitivity of 86.6% and a specificity of 37.7% (AUC: 0.719, 95% CI (0.625 - 0.813; P < 0.001).

### Levels of serum cytokines predicts functional outcome

167 patients completed the 3-month follow-up. Poor prognosis was found in 54 (32.3%) patients with 27 of them (50.0%) being male and mainly occurred in older ages and patients with a history of smoking. We found that serum IL-4 level was significantly lower in AIS patients with poor outcome compared with those with a good prognosis 3.10 (IQP 1.13 - 8.15) pg/mL *vs.* 0.53 (IQR 0.28 - 1.32) pg/mL, P < 0.001), IL-5 level also significantly decreased in the poor outcome group [0.63 (IQR 0.48 - 0.86) pg/mL *vs.* 0.32 (IQR, 0.24 - 0.66) pg/mL, P < 0.001] (Fig. 2). Serum IL-6 was significantly higher in the poor outcome group (Table 3) while serum levels of IL-1β, IL-7, IL-9, IL-10, IL-15, G-CSF, GM-CSF remained relatively constant.

By univariate logistic regression analysis, we found that the serum concentrations of neutrophils, IL-4, IL-5, IL-6, and MIP-1α were significantly associated with functional outcome of AIS patients (Table 3). Multivariate logistic regression was used to further analysis these parameters in unadjusted models (including age, gender, SBP, DM, cardiac disease, smoking, alcohol drinking, WBCs, neutrophils, serum levels of IL-4, IL-5, IL-6, MDC and MIP-1α, infract volume, NIHSS scores on admission and on discharge, the use of antiplatelet agents and statin). We found that serum concentration of IL-5 functioned as a protective factor and was an independent predictor for functional outcome of AIS patients with an adjusted OR of 0.039 (95% CI, 0.003 - 0.475, P = 0.011), whereas IL-6 was an independent risk factor in functional outcome with an adjusted OR of 1.329 (95% CI,1.095 - 1.612, P = 0.004). Furthermore, NIHSS score on discharge has alsobeen used as an indpdendent predictor of AIS patient outcome. Based on the ROC curve analysis, the optimal cutoff value of IL-5 for predicting AIS prognosis was projected to be 0.385 pg/mL (with a sensitivity of 86.6% and a specificity of 37.7%) and AUC: 0.719, 95% CI (0.625 - 0.813, P < 0.001, Fig. 3).

## DISCUSSION

In this study, we found that IL-1RA, IL-1β, IL-4, IL-5, IL-6, IL-7, IL-9, IL-10, IL-13, IL-15, EGF, G-CSF, Flt-3L, GM-CSF and Fractalkine levels were significantly decreased in the severe stroke group. While in AIS patients with poor outcome, IL-1β, IL-4, IL-5, IL-7, IL-9, IL-10, IL-15, G-CSF and GM-CSF were significantly reduced. We also found that IL-5 was an independent protective factor for prognosis and IL-6 was an independent risk predictor for AIS patients. Our data suggest that the levels of serum cytokines were related to stroke severity, short-term prognosis and cerebral infarct volume of AIS patients.

A large body of evidence suggests that inflammation is a major contributor to the pathophysiologic processes of ischemic stroke. The intimate balance between pro- and anti-inflammatory cytokines are relevant to the susceptibility and functional outcome of patients with AIS [5, 37]. IL-4 is a product of selective immune cells, acting as a pleiotropic regulator of numerous immune and inflammatory responses [38, 39]. It has been confirmed that IL-4 could polarize macrophages from a proinflammatory M1 phenotype to a “healing” M2 phenotype, which would play an anti-inflammatory role in tissue repair [40, 41]. Zhao et al. reported that IL-4 secreted by neuronal cells in ischemia acts as a neuroprotective mechanism to aid in the regulation of intracerebral cleanup and repair after stroke [42]. IL-5, a primary T-cell-derived cytokine, regulates eosinophil development and is regarded as a significant contributor to atopic diseases such as asthma [43, 44]. An increasing number of studies support the idea that IL-5 induces the production of anti-Ox-LDL antibodies and plays an anti-atherosclerotic role by enhancing Th2-type immune responses [45, 46]. Sämpi *et al*. suggested that IL-5 level was related to antibodies binding to the Ox-LDL and inversely connected with carotid intima-media thickness [47]. Studies have found that the expression levels of IL-4, IL-5, IL-6, and IL-10 are increased remarkably in the damaged hemisphere after ischemic stroke. IL-4 is mostly known as an anti-inflammatory cytokine and IL-5 have been shown to suppress post-stroke inflammation [16]. In our study, we found that serum concentrations of IL-4, IL-5 and IL-9 were significantly elevated in minor stroke patients and negatively related to the NIHSS score. These biomarkers were also associated with functional outcome of AIS patients and markedly decreased in AIS patients with a poor prognosis. Moreover, we showed that IL-5 possessed a protective role and was an independent factor that could predict AIS patients’ prognosis. Thus, we speculate that IL-4, IL-5 and IL-9 could act as protective factors for AIS. In fact, Xiong et al. demonstrated that IL-4 was somehow involved in cerebral ischemic outcome as IL-4 deficiency resulted in a greater degree of ischemic brain damage [15]. Similarly, Sheikh et al. demonstrated that pretreatment of HMO6 cells with IL-5 could suppress the expression of IL-1β and IFNγ-induced mRNA, and IL-5 had the capability to inhibit focal ischemia-induced inflammation [17]. Therefore, the regulation of IL-4 or IL-5 and its associated pathways are potential targets for the treatment of cerebral ischemia. As a pro-inflammatory cytokine, IL-9 can promote CCL20 release from astrocytes and the migration of Th17 cells into the central nervous system [48]. The mechanisms underlying IL-9 and Th9 cells-mediated ischemic injury are largely unknown. Like TNF-α and IL-1β, IL-9 may directly damage brain tissue as they perpetuate pro-inflammatory actions [49, 50]. Tan et al. demonstrated that the expression level of IL-9 and percentages of Th9 and Tc9 cells were notably higher in (PBMCs) derived from ischemic stroke patients. Moreover, an increased level of IL-9 may compromise the BBB’s integrity through IL-9R/STAT1,3 pathways [51], but this was negative correlated with stroke severity.

Notably, there was a controversy regarding the source of elevated IL-6 levels in the early stroke phase [52]; IL-6 possessed multipotent functions and was upregulated during brain injury or the repair process [53]. IL-6 plays a dual role in the inflammatory response induced by cerebral ischemia. On one hand, IL-6 would further aggravate the deterioration of brain damage and counteract neural stem cell proliferation in the acute phase of cerebral ischemia. On the other hand, IL-6 would restore and enhance glial cells to prevent collagen deposition and repair brain damage in the subacute phase [54]. Previous studies reported that circulating IL-6 was closely correlated with brain infarct volume or stroke severity [53, 55], although contradicting results were reported [25]. Besides, Smith et al. revealed that peak plasma IL-6 level in stroke patients was correlated with poor prognosis [55]. A recent report recommended that IL-6 induced by IL-1 was associated with worse prognosis in stroke patients, and that clinical outcome would be improved after the inflammatory factor was reduced by IL-1 receptor antagonist [56]. Consistently, our study showed that IL-6 level was significantly elevated in AIS patients with poor outcome. However, Karen et al. suggested that IL-6 is beneficial for long-term prognosis as IL-6 could promote early transcriptional changes in angiogenesis-related genes after cerebral ischemia, which afford long-term histological and functional protection [57]. Therefore, this phenomenon needs to be further studied. We found that the level of serum IL-7 was notably reduced in patients with severe stroke and poor outcome. As a pleiotropic cytokine, IL-7 is capable of multiple effects [58]. IL-7 could act as a regulator of growth or anti-apoptosis in pre-B cells, and maintain the steady state proliferation of mature T cells, which might effectively reduce neuroinflammatory and some autoimmune responses through the inhibition of signal transduction [20, 59]. Cagnin and Damas reported that IL-7 was upregulated in patients with cardiovascular atherosclerosis (particularly among patients with acute myocardial infarction and angina pectoris) compared with controls [60, 61]. Controversially, other studies showed that IL-7 level was significantly reduced in stroke patients compared to controls [62]. Lawson et al. speculated that IL-7 signaling was a prerequisite for activating optimal CD4^+^ T cell and that IL-7R antagonism could be an effective treatment for CD4^+^ T cell-mediated neuroinflammation and several inflammatory autoimmune diseases [59].

Here, we also found that levels of IL-1β, IL-1RA, IL-10, IL-13, IL-15, Flt-3L, Fractalkine, EGF, G-CSF and GM-CSF were lower in the severe stroke group. IL-1β is an important mediator of the inflammatory response and involved in a variety of cellular activities, including cell proliferation, differentiation, and apoptosis. Several studies found that IL-1β were elevated in the AIS group [63, 64]. Similarly, IL-1RA levels were found to be elevated in stroke patients in previous studies [65, 66]. IL-10 is an anti-inflammatory cytokine produced by T cells and monocytes, suggesting its participation in vascular protection, although the exact mechanisms are unclear. In an animal model, cerebral infarct volume have been reduced through intraventricular or systemic administration of IL-10 [67], and IL-10 has been suggested to be neuroprotective [67, 68]. IL-13 is a cytokine mainly secreted by Th2 cells [22]. The secondary structural features of IL-13 are like IL-4 and share similar functions with IL-4. IL-13 possess anti-inflammatory properties and is mainly associated with diseases involving the airway. However, there are few reports regarding IL-13’s participation in stroke. IL-15 is a cytokine with structural similarity to IL-2. Lee et al. found that IL-15 promote astrocyte survival in response to OGD, thus can be beneficial to ischemic stroke [69]. However, there is no clinical data showing a link between IL-15 and stroke. Flt3L is a growth factor, which can stimulate not only the proliferation and differentiation of hematopoietic progenitor cells, leading to increased numbers of pre-B cells, but also the maturation of T, B and NK cells [70, 71]. Fractalkine was initially discovered as an adhesion molecule for lymphocytes and monocytes, natural killer cells, and microglia, indicating its role for regulating the inflammatory response [72]; Fractalkine can also decrease microglial activation and release pro-inflammatory cytokines [73, 74]. EGF, G-CSF and CM-CSF are trophic factors and capable of neuroprotection. Administration of EGF and G-CSF in a rat MCAO model could reduce infarct volume [75, 76]. Navarro et al. found that the level of GM-CSF was significantly higher in stroke patients than in healthy controls, and was positively correlated with NIHSS score [77].

MDC is a member of the CC-chemokine family and is mainly produced by macrophages and dendritic cells. There is an increasing number of reports regarding the involvement of MDC in a variety of diseases, ranging from allergic reactions to HIV infection and neoplasia [78]. Kimura et al. suggested that MDC promotes atherosclerosis by migration or recruitment of monocyte-derived cells and the stimulation of platelet activity [79]. MIP-1α belong to the CC-chemokine subfamily, possessing inflammatory and neutrophil chemokinetic properties [80]. Gourmala et al. revealed an early increase in macrophage inflammatory protein-1α and macrophage inflammatory protein-1β messenger RNA levels in a rat MCAO model [81].

Several limitations in the study should not be neglected. First, this is a single center study with a small number of patients. As stroke patients are mostly elderly persons, it was difficult to find age-matched healthy subjects. Second, serum cytokine levels were tested only at admission. Monitoring the dynamic change of serum biomarker levels will be essential in further studies. Last, a minor stroke is generally defined as a NIHSS of 5 or less, which only considers certain deficits but not the fact that some patients can have a more profound impact on the quality of life versus others. Therefore, the scale does not linearly correlate deficit and disability. Recent studies suggest that score of an NIHSS of 3 or less may be a better definition of a minor stroke [82, 83].

In conclusion, the present study is the first report to demonstrate the simultaneous measurement of 35 serum cytokines, chemokines, and growth factors in patients with AIS. We found that the serum concentration of these factors is significantly associated with the outcome for the AIS patient. Indeed, an increased knowledge of inflammatory mediators in response to AIS may provide a basis for the design and development of new pharmacological approaches to treat stroke.
